# Predictors for health-related quality of life in patients with rheumatoid arthritis: a longitudinal study

**DOI:** 10.1093/rap/rkaf116

**Published:** 2025-10-09

**Authors:** Hermann Szymczak, Susanne Brandstetter, Boris Ehrenstein, Mark Steinmann, Christian Apfelbacher

**Affiliations:** Institute of Social Medicine and Health Systems Research, Medical Faculty, Otto von Guericke University Magdeburg, Magdeburg, Germany; University Children’s Hospital Regensburg (KUNO), University of Regensburg, Hospital St Hedwig of the Order of St John, Regensburg, Germany; Medical Sociology, Institute for Epidemiology and Preventive Medicine, University of Regensburg, Regensburg, Germany; Department of Rheumatology and Clinical Immunology, Asklepios Medical Center Bad Abbach, Bad Abbach, Germany; DONAUISAR Klinikum Deggendorf, Deggendorf, Germany; Institute of Social Medicine and Health Systems Research, Medical Faculty, Otto von Guericke University Magdeburg, Magdeburg, Germany; Medical Sociology, Institute for Epidemiology and Preventive Medicine, University of Regensburg, Regensburg, Germany

**Keywords:** beliefs about medicines, health-related quality of life, medication adherence, rheumatoid arthritis, longitudinal study

## Abstract

**Objectives:**

Existing observational research on predictors of health-related quality of life (HRQoL) in patients with RA is mostly limited to cross-sectional designs and often focuses on one or a few predictors. This is the first comprehensive analysis of a set of selected predictors in a longitudinal setting.

**Methods:**

A total of 361 individuals with RA were included in the study. HRQoL (physical and mental component summary scales of the 12-item Short Form Health Survey) was assessed at baseline, 3- and 12-month follow-ups. In total, 19 predictors were assessed at baseline, grouped into six thematic blocks: demographics (age, sex, education), disease-related factors (duration of disease, pain, illness severity), therapy-related factors (side effects, number of RA medicines, type of prescribed RA medication), psychological variables (beliefs about medicines, social support, depression, anxiety), behaviour (adherence) and lifestyle (obesity). Hierarchical multiple linear regression analysis was employed to test the influence of individual (groups of) predictor variables on HRQoL.

**Results:**

Only pain (as a disease-related factor) predicted both physical and mental HRQoL at all follow-ups. For physical HRQoL, age was a significant predictor at both follow-ups, as were anxiety and depression (psychological variables) for mental health HRQoL. For all other included variables, findings were non-significant or inconsistent across measurement points.

**Conclusion:**

Even though the selection of possible predictors was based on previous research findings, analysis revealed that the impact may not be as robust as previously thought. More longitudinal research is needed to specify factors that impact HRQoL in RA patients in the long run.

Key Messages

- Pain predicts both physical and mental health-related quality of life (HRQoL) in RA patients.
- Age predicts physical HRQoL, while anxiety and depression predict mental HRQoL.
- Predictors selected based on previous research may have a weaker impact than previously thought.

## Introduction

RA is a chronic inflammatory disease characterized by pain and swelling of the joints leading to disability and reduced quality of life (QoL) [1, 2]. Significant advancements in medical treatment, particularly following the advent of biologic therapies in the 1990s, have resulted in enhanced disease control, reduced joint damage and improved QoL for a considerable proportion of patients. The primary objectives of contemporary therapeutic interventions are now focused on achieving remission/low disease activity, effective symptom management, the maintenance of physical function and optimizing health-related quality of life (HRQoL).

Consequently, HRQoL has emerged as a pivotal patient-reported outcome measure (PROM) in RA research. However, existing research on predictors of HRQoL in RA patients is often limited, as it mostly focuses on one or only a few specific predictors, such as adherence to medication, fatigue or comorbidities; and is primarily cross-sectional [e.g. 3–5]. For instance, Wan *et al.* [6] found in a cross-sectional study with 108 outpatients that pain, functional disability and depression were main predictors of HRQoL. In a recent study, Torlinska *et al.* [7] analysed data of 484 RA patients in the Birmingham Early Arthritis Cohort and found that HRQoL was associated with disease activity, polypharmacy, obesity, sex and older age. In one of the few longitudinal analyses investigating predictors of HRQoL, non-adherence to methotrexate was significantly associated with lower HRQoL during a 4-year follow-up of RA patients [8]. Cohen *et al.* [9] conducted a 5-year prospective cohort study with several possible predictors for disease-specific QoL in early RA measured by the AIMS2, including age, sex, BMI, disease duration, DAS, HAQ and various biological markers, and found in their multivariate analyses that the baseline HAQ score was the best predictive factor.

The present study aims to add to the extant literature by analysing a comprehensive range of preselected determinants, encompassing sociodemographic, disease-related, therapy-related, psychological and behavioural factors, on both physical and mental HRQoL of RA patients.

## Methods

### Study design and sample

The study was conducted at the Department of Rheumatology, Asklepios Medical Center Bad Abbach, a tertiary care centre for patients with autoimmune and rheumatic diseases, located in Bavaria, Germany [10, 11]. At the initial study visit [baseline (T0)], participants were asked to complete self-report questionnaires. In addition, information was extracted from electronic health records. Patients were eligible for participation if they were ≥18 years of age, had physician-diagnosed RA and were currently under medical treatment for RA. Baseline data were collected in 2012 and 2013 (T0, *n* = 361). Two follow-ups were conducted by mail, 3 (T1, *n* = 292) and 12 (T2, *n* = 245) months later. In case of non-response, up to two reminder mails were sent to participants. The study has been approved by the ethics committee of the University of Regensburg (file number: 12-101-0126). Written informed consent to participate was obtained from 361 participants.

The reporting of this study follows the Strengthening the Reporting of Observational Studies in Epidemiology (STROBE) reporting guidelines [12]. The STROBE checklist for this publication is available as Supplementary Data S1.

### Measures

#### Predictors

Included predictor variables were chosen based on previous literature, capturing individual, psychological and behavioural influences. Predictors are described in Table 1. For the purpose of clarity, predictors are classified by theme (and thus according to their entry in the hierarchical regression model).

**Table 1.** Overview of predictor variables and assessment method, classified by six thematic blocks of the regression model

Block	Theme (number of variables)	Variables/predictor	Variable description
1	Demographic (3)	Age, sex, education	Basic demographic data: age (years), sex (male/female), education (schooling ≥10 years; yes/no)
2	Disease related factors (3)	Duration of disease	Years since diagnosis.
		Pain	Self-rated pain (VAS, range 1–10)
		Illness severity	DAS28 [13, 14]
3	Therapy related factors (3)	Side effects	Item: ‘Have you ever experienced serious side effects from the medication you are taking to treat your rheumatic disease?’ (yes/no)
		Number of RA medicines	Total prescribed RA medicines according to medical records
		Type of prescribed RA medication	Biologics (yes//no)
4	Psychological variables (8)	Beliefs about medicines: five scales	BMQ [15, 16]; 23-item questionnaire with five scales: general beliefs: overuse, harm, utility; specific beliefs: necessity, concerns
		Social support	Social Support Questionnaire (F-SozU K-14 [17])
		Depression, anxiety	HADS [18]
5	Behaviour (1)	Adherence	Medication Adherence Report Scale (MARS) [19]: dichotomized: adherence *vs* non-adherence
6	Lifestyle (1)	Obesity	BMI ≥30 (yes/no)

All predictors were assessed at baseline.

VAS: Visual Analogue Scale.

The sequence of variable entry was determined based on a comprehensive literature review and expert consensus among our research team. The hierarchical approach was chosen to systematically assess the unique contribution of different variable categories to HRQoL. The overall strategy was to introduce variables in a logical order, moving from broad control variables to factors with increasing proximity to the disease itself, followed by more distal and exploratory factors. The rationale for the specific order of entry is as follows: Demographics were entered first as foundational control variables. Following demographics, the variables were entered in descending order of proximity to the disease. Therefore, disease- and therapy-related factors, which have a more direct and proximal effect on the physiological condition and HRQOL, were entered second and third, respectively. Next, psychological variables were introduced, as they represent a strong but comparatively more distal component of RA. Behaviour was considered an even more distant factor and was entered late to assess the additional variance it might explain after accounting for all other factors. Finally, lifestyle was entered as an exploratory variable to determine its unique contribution to the model.

#### Outcome: HRQoL

The 12-item Short Form Health Survey (SF-12) was used to measure HRQoL [20, 21]. Scores for the physical and mental component summaries of the SF-12 (PCS-12, MCS-12) range from 0 to 100 (higher values indicate better HRQoL) and are normalized for the general German population, resulting in a standard score with a mean of 50 (s.d. 10) [22, 23].

### Statistical analyses

All statistical analyses were prespecified in a statistical analyses plan. In the context of continuous variables, either the median and interquartile range (IQR) or the mean and s.d. are reported. For categorical variables, counts and percentages were employed. Group differences were determined by *t*-tests and bivariate associations between variables via correlations.

Hierarchical linear regression analysis was employed to test the influence of individual (groups of) predictor variables on the outcome [24, 25]. In total, 19 variables were selected before the beginning of the study and are grouped into six thematic blocks (resulting in six corresponding regression steps; see Table 1). To identify potential multicollinearity, bivariate correlations between all predictors are investigated as well as variance inflation factors (VIFs). A correlation (*r*) >0.8 and VIF >10 are considered of concern. To avoid overfitting, a minimum of 10–15 events per variable is required [26, 27]. For our 19 variables we should therefore have 190–285 observations. The final models had 224 (3 months) and 186 (12 months) observations (cf. Results section). Model fit is reported as adjusted *R*^2^ and model improvement between hierarchical steps as Δ*R*^2^. Results for individual predictors of the final model are given as beta values. *P*-values <0.05 were interpreted as statistically significant. All analyses were performed using SPSS 29 (IBM, Armonk, NY, USA).

## Results

### Sample characteristics and predictor variables

The characteristics of the study participants are detailed in Table 2. Approximately 30% of the participants were male, with an average age of 60 years (s.d. 13.4). The average duration of RA since diagnosis among participants was 11 years (s.d. 9.2).

**Table 2.** Sociodemographic, psychological and medical characteristics of study participants at baseline (T0, *n* = 361).

Variable	*n*	Result
Age, years, mean (s.d.)^a^	359	60.2 (13.36)
Male, *n* (%)^a^	361	110 (30.5)
Education ≥10 years (%)^a^	361	39.4
Duration of disease, months, mean (s.d.)^a^	330	10.94 (9.22)
Age at diagnosis, years, mean (s.d.)	331	49.26 (15.21)
Migration background, %	361	7.2
Inpatient treatment, %	360	21.7
Distance to provider of RA treatment, km, mean (s.d.)	357	58.59 (44.75)
Self-rated pain (1–10), mean (s.d.)^a^	359	4.47 (2.78)
DAS28, mean (s.d.)^a^	361	3.07 (1.57)
Experience of severe side effects, yes, %^a^	361	43.2
Number of prescribed medicines, mean (s.d.)	360	7.13 (3.68)
Number of prescribed RA medicines, mean (s.d.)^a^	360	3.86 (1.60)
Biologics (any), %^a^	360	29.2
NSAR (any), %	360	16.4
Opioids (any), %	360	14.4
Corticosteroids (any), %	360	71.9
DMARDs (any), %	360	75.8
BMQ general–overuse, mean (s.d.)^a^	354	3.14 (0.79)
BMQ general–harm, mean (s.d.)^a^	351	2.53 (0.75)
BMQ general–utility, mean (s.d.)^a^	349	3.97 (0.64)
BMQ specific–necessity, mean (s.d.)^a^	355	4.27 (0.64)
BMQ specific–concerns, mean (s.d.)^a^	348	2.84 (0.80)
F-SozU K-14, mean (s.d.)^a^	357	4.11 (0.74)
HADS depression, mean (s.d.)^a^	354	6.03 (3.83)
Clinically significant depression score, *n* (%)^b^		55 (15.2)
HADS anxiety, mean (s.d.)^a^	352	7.13 (3.89)
Clinically significant anxiety score, *n* (%)^b^		69 (19.1)
MARS, % non-adherence, mean (s.d.)^a^	351	68
BMI, mean (s.d.)	357	27.75 (5.80)
Obesity (BMI ≥30), *n* (%)^a^	357	109 (30.5)

NSAR: non-steroidal anti-inflammatory drug; F-SozU K-14: Social Support Questionnaire; MARS: Medication Adherence Report Scale (dichotomized: adherence *vs* non-adherence).

a Predictor for regression model.

b HADS cut-off value at 11 for indication of clinically significant mood disorder [18].

### Descriptive results for main outcome (HRQoL)

PCS and MCS values of participants were below the population mean of 50 and remained constant between measurement points, with MCS being somewhat higher than PCS [PCS values: mean 36.5 (s.d. 10.4), 37.2 (s.d. 10.9), 37.3 (s.d. 10.4); MCS values: mean 42.1 (s.d. 13.6), 42.6 (s.d. 13.1), 41.7 (s.d. 13.0) at baseline (T0), 3 months (T1) and 12 months (T2) follow-up, respectively]. The rate of non-completed PCS/MCS measures for each measurement point was 7% (*n* = 335 of total, *n* = 361 at T0; *n* = 272 of total, *n* = 292 at T1; and *n* = 227 of total, *n* = 245 at T2; see Supplementary Figure S1, which shows the histogram for PCS and MCS at 3 and 12 months follow-up).

### Regression analyses

Overall results for all regression models (adjusted *R*^2^ and Δ*R*^2^) are reported in Table 3 (PCS) and Table 4 (MCS) [variables added: demographic variables, disease-related factors, therapy-related factors, psychological variables, behaviour (adherence) and lifestyle (obesity)]. Multicollinearity analysis shows that none of the bivariate correlations between predictors was *r* >0.8 {highest correlations were between Hospital Anxiety and Depression Scale [HADS] depression and HADS anxiety [*r* = 0.668] and pain and illness severity [28-item DAS (DAS28; *r* = 0.641)]}. The maximum VIF across the final models was 2.3–2.8 (HADS depression), 2.2–2.5 (HADS anxiety), 1.9–2.2 (pain) and 2.0–2.2 [illness severity (DAS28)]. Thus all VIFs were ≤2.8 and therefore were not considered of concern with regard to multicollinearity.

**Table 3.** Hierarchical regression model summary for PCS-12 at the 3- and 12-month follow-up.

Model	Adjusted *R*^2^	SE	Δ*R*^2^	df1	df2	F-change	F-change *P*-value
PCS-12 at 3 months (*n* = 224)							
1 Demographics	0.119	10.13	**0.131**	3	220	**11.052**	**<0.001**
2 Disease-related factors	0.329	8.84	**0.216**	3	217	**23.901**	**<0.001**
3 Therapy-related factors	0.379	8.50	**0.058**	3	214	**6.890**	**<0.001**
4 Psychological variables	0.394	8.40	0.036	8	206	1.662	0.110
5 Behaviour (adherence)	0.393	8.41	0.001	1	205	0.443	0.507
6 Lifestyle (obesity)	0.418	8.24	**0.026**	1	204	**9.770**	**0.002**
PCS-12 at 12 months (*n* =186)							
1 Demographics	0.117	9.87	**0.132**	3	182	**9.195**	**<0.001**
2 Disease-related factors	0.296	8.81	**0.188**	3	179	**16.448**	**<0.001**
3 Therapy-related factors	0.308	8.74	0.022	3	176	1.998	0.116
4 Psychological variables	0.323	8.65	0.043	8	168	1.482	0.167
5 Behaviour (adherence)	0.319	8.67	0.000	1	167	0.127	0.722
6 Lifestyle (obesity)	0.330	8.60	0.013	1	166	3.645	0.058

*P*-values for F-change bold if <0.05.

Model steps: hierarchical inclusion from 1 to 6.

SE: standard error of the estimate.

**Table 4.** Hierarchical regression model summary for MCS-12 at the 3- and 12-month follow-up.

Model	Adjusted *R*^2^	SE	Δ*R*^2^	df1	df2	F-change	F-change *P*-value
MCS-12 at 3 months (*n* = 224)							
1 Demographics	0.029	13.07	**0.042**	3	220	**3.187**	**0.025**
2 Disease-related factors	0.104	12.56	**0.086**	3	217	**7.159**	**<0.001**
3 Therapy-related factors	0.103	12.56	0.011	3	214	0.933	0.426
4 Psychological variables	0.489	9.48	**0.389**	8	206	**21.186**	**<0.001**
5 Behaviour (adherence)	0.488	9.49	0.002	1	205	0.730	0.394
6 Lifestyle (obesity)	0.488	9.49	0.003	1	204	1.101	0.295
MCS-12 at 12 months (*n* = 186)							
1 Demographics	0.012	12.94	0.028	3	182	1.766	0.155
2 Disease-related factors	0.114	12.26	**0.114**	3	179	**7.938**	**<0.001**
3 Therapy-related factors	0.118	12.23	0.018	3	176	1.279	0.283
4 Psychological variables	0.414	9.97	**0.307**	8	168	**12.122**	**<0.001**
5 Behaviour (adherence)	0.411	9.99	0.001	1	167	0.187	0.666
6 Lifestyle (obesity)	0.408	10.02	0.001	1	166	0.181	0.671

*P*-values for F-change bold if *P* < 0.05.

Model steps: hierarchical inclusion from 1 to 6.

SE: standard error of the estimate.

In total, ≈30–50% of variance in HRQoL in our data was explained in the final models, both for PCS and MCS (adjusted *R*^2^ = 0.33–0.49; Tables 3 and 4). Disease-related factors (duration of disease, pain, illness severity) explained additional variance in HRQoL in the PCS and MCS at all follow-ups (Δ*R*^2^ = 0.09–0.22, *P* < 0.001). For PCS, demographic variables were significant contributors at both the 3- and 12-month follow-up (Δ*R*^2^ = 0.13, *P* < 0.001), whereas therapy-related factors and lifestyle were only significant at the 3-month follow-up (Δ*R*^2^ = 0.06 and 0.03, *P* < 0.01). For MCS, psychological variables were the strongest predictors at both the 3 and 12-month follow-ups (Δ*R*^2^ = 0.31–0.39, *P* < 0.001). Demographic variables predicted MCS only marginally at the 3-month follow-p (Δ*R*^2^ = 0.04, *P* = 0.025).

Parameters for the individual predictors (only for the final regression model) are presented in Table 5  **(**PCS at 3 and 12 months) and Table 6 (MCS at 3 and 12 months). Looking at individual predictors for PCS (Table 5), age and pain (at baseline) were significant predictors for HRQoL at both 3 and 12 months. Interestingly, disease activity and side effects of medications were only significant predictors for HRQoL at 3 months.

**Table 5.** Hierarchical regression of PCS-12 at the 3- and 12-month follow-up (final model).

Predictor	*B*	β	*t*	*P*-value
PCS-12 at 3 months (final model, *n* = 224)				
Age (years)	−0.213	**−0.255**	−4.329	**<0.001**
Sex^a^	0.618	0.027	0.473	0.637
Education^b^	−1.571	−0.072	−1.287	0.200
Duration of disease	−0.080	−0.071	−1.261	0.209
VAS pain	−0.784	**−0.196**	−2.744	**0.007**
DAS28	−1.331	**−0.190**	−2.654	**0.009**
Side effects^c^ (yes/no)	−3.255	**−0.149**	−2.589	**0.010**
Prescribed RA medication	−0.522	−0.075	−1.281	0.202
Biologicals yes/no	−1.587	−0.067	−1.198	0.232
BMQ general–overuse	0.868	0.063	0.941	0.348
BMQ general–harm	−1.401	−0.090	−1.264	0.208
BMQ general–utility	0.257	0.015	0.247	0.806
BMQ specific–necessity	−2.333	**−0.136**	−2.257	**0.025**
BMQ specific–concern	1.323	0.092	1.284	0.201
F-SozU (social support)	0.289	0.019	0.292	0.770
HADS depression	−0.372	−0.128	−1.485	0.139
HADS anxiety	0.020	0.007	0.090	0.928
Adherence (yes/no)	1.636	0.071	1.271	0.205
Obesity (yes/no)	−3.817	**−0.168**	−3.126	**0.002**
PCS-12 at 12 months (final model, *n* = 186)				
Age (years)	−0.255	**−0.302**	−4.281	**<0.001**
Sex^a^	0.078	0.003	0.052	0.959
Education^b^	−1.394	−0.065	−0.994	0.321
Duration of disease	−0.154	**−0.139**	−2.075	**0.040**
VAS pain	−0.915	**−0.239**	−2.701	**0.008**
DAS28	−0.814	−0.116	−1.308	0.193
Side effects^c^ (yes/no)	−2.596	−0.123	−1.726	0.086
Prescribed RA medication	−0.331	−0.050	−0.740	0.461
Biologics (yes/no)	−1.017	−0.044	−0.660	0.510
BMQ general–overuse	−1.445	−0.112	−1.396	0.165
BMQ general–harm	−0.368	−0.026	−0.294	0.769
BMQ general–utility	2.177	0.139	1.925	0.056
BMQ specific–necessity	−1.250	−0.077	−1.079	0.282
BMQ specific–concern	0.939	0.069	0.816	0.415
F-SozU (social support)	−0.382	−0.026	−0.353	0.725
HADS depression	−0.322	−0.114	−1.195	0.234
HADS anxiety	0.461	**0.179**	1.985	**0.049**
Adherence (yes/no)	1.096	0.049	0.726	0.469
Obesity (yes/no)	−2.720	−0.122	−1.909	0.058

SES: socio-economic status; SSS: subjective social status.

*P*-values bold if <0.05.

a Sex: 0, male; 1, female.

b Education: school education dichotomized (0, low; 1, high).

c Ever experienced severe side effects from medication.

**Table 6.** Hierarchical regression of MCS-12 at the 3- and 12-month follow-up (final model).

Predictor	*B*	β	*t*	*P*-value
MCS-12 at 3 months (final model, *n* = 224)				
Age (years)	−0.001	−0.001	−0.011	0.991
Sex^a^	1.307	0.046	0.869	0.386
Education^b^	0.859	0.032	0.611	0.542
Duration of disease	−0.009	−0.006	−0.121	0.904
VAS pain	−0.833	−0.169	**−2.529**	**0.012**
DAS28	0.280	0.032	0.485	0.628
Side effects^c^ (yes/no)	2.021	0.075	1.396	0.164
Prescribed RA medication	0.167	0.019	0.356	0.722
Biologics (yes/no)	−0.297	−0.010	−0.194	0.846
BMQ general–overuse	0.040	0.002	0.038	0.970
BMQ general–harm	−1.097	−0.057	−0.860	0.391
BMQ general–utility	0.327	0.016	0.273	0.785
BMQ specific–necessity	−0.308	−0.015	−0.259	0.796
BMQ specific–concern	−0.015	−0.001	−0.013	0.990
F-SozU (social support)	−0.240	−0.013	−0.210	0.834
HADS depression	−1.330	−0.372	**−4.614**	**<0.001**
HADS anxiety	−1.290	−0.381	**−5.037**	**<0.001**
Adherence (yes/no)	−1.544	−0.055	−1.041	0.299
Obesity (yes/no)	1.476	0.053	1.049	0.295
MCS-12 at 12 months (final model, *n* = 186)				
Age (years)	−0.010	−0.009	−0.140	0.889
Sex^a^	0.808	0.029	0.458	0.647
Education^b^	1.204	0.046	0.738	0.462
Duration of disease	−0.062	−0.046	−0.722	0.471
VAS pain	−0.984	**−0.208**	−2.496	**0.014**
DAS28	0.475	0.055	0.656	0.513
Side effects^c^ (yes/no)	0.855	0.033	0.488	0.626
Prescribed RA medication	0.231	0.028	0.442	0.659
Biologics (yes/no)	0.444	0.015	0.247	0.805
BMQ general–overuse	0.354	0.022	0.294	0.769
BMQ general–harm	−1.574	−0.091	−1.079	0.282
BMQ general–utility	−1.475	−0.076	−1.121	0.264
BMQ specific–necessity	0.137	0.007	0.101	0.919
BMQ specific–concern	−0.478	−0.028	−0.357	0.722
F-SozU (social support)	−1.647	−0.089	−1.305	0.194
HADS depression	−1.266	**−0.363**	−4.035	**<0.001**
HADS anxiety	−1.040	**−0.326**	−3.846	**<0.001**
Adherence (yes/no)	−0.891	−0.032	−0.507	0.613
Obesity (yes/no)	0.705	0.026	0.425	0.671

*P*-values bold if <0.05.

a Sex: 0, male; 1, female.

b Education: school education dichotomized (0, low; 1, high).

c Ever experienced severe side effects from medication.

SES: socio-economic status; SSS: subjective social status; F-SozU K-14: Social Support Questionnaire.

Looking at individual factors for MCS (Table 6), pain, depression and anxiety were significant predictors at the 3- and 12-month follow-up (β = −0.38 to −0.17, *P* < 0.05).

## Discussion

### Key findings

Of all the variables included in the study, only pain, as a disease-related factor, predicted both physical and mental HRQoL at all follow-ups. For physical HRQoL, age was a significant predictor at both follow-ups, as were anxiety and depression (psychological variables) for mental HRQoL. For all other included variables, the findings were non-significant or inconsistent across measurement points.

### Interpretation in relation to the literature

As has been demonstrated in previous literature, HRQoL scores in our sample were considerably diminished when compared with the general population, with the physical domain being more significantly affected than the mental [28]. Matcham *et al.* [29] found in their review and meta-analysis on the impact of RA on QoL assessed using the SF-36, including >22 000 patients, that the pooled mean HRQoL score for the SF-36 PCS was 34.1 (95% CI 22.0, 46.1) and for MCS was 45.6 (95% CI 30.3, 60.8). These findings are consistent with the results of the present study.

The present findings underscore the pivotal function of pain in HRQoL for individuals with RA, a conclusion that is corroborated by extant research. Pain is frequently cited as the primary concern by RA patients and constitutes a key treatment focus (e.g. Di Matteo et al. [2]). Moreover, the influence of age on physical HRQoL aligns with previous research as well as the robust negative correlation between anxiety and depression with mental HRQoL [e.g. Haridoss *et al.* [30]). In this respect it is important to note that depression is the most common psychiatric comorbidity in RA [31, 32], and anxiety is also highly prevalent in this population [33–35]. The findings on pain, age, depression and anxiety, as a whole, corroborate earlier research and underscore the predictive validity and significance of these factors.

Findings for all other investigated variables are inconclusive, i.e. these were not found consistently at both the 3- and 12-month follow-up for PCS and MCS, respectively. This may be partly due to the longitudinal character of the present study, as the strengths of associations might diminish over time and/or the predictor variables themselves have changed. For instance, the adjusted *R*^2^s of the final models were considerably smaller after 12 months than after 3 (PCS: *R*^2^ = 0.42–0.33, Table 4; MCS: *R*^2^ = 4.9–4.1). For PCS, disease activity, side effects of RA medication, Beliefs about Medicines Questionnaire (BMQ) scale ‘necessity’ and obesity were significant predictors at the 3-month follow-up (in addition to pain and age). However, at the 12-month follow-up, no significant predictors emerged, although anxiety and the duration of the disease became significant at this point. For MCS, the findings were more consistent, with only pain, depression and anxiety scores remaining significant at all points.

Surprisingly, we found no significant association between adherence to RA medication and HRQoL. One potential explanation lies in the hierarchical modelling approach, where adherence was entered last after accounting for other variables, potentially diminishing its apparent effect. However, bivariate analyses revealed no predictive relationship between adherence at baseline (T0) and physical HRQoL at 3 or 12 months (*t*[264] = 0.559, *P* = 0.576; *t*[221] = −0.263, *P* = 0.793). In contrast, adherence at T0 did predict mental HRQoL at both the 3-month (*t*[264]  = −3.081, *P* < 0.01) and 12-month follow-ups (*t*[221] = −3.5, *P* < 0.001). It is also important to stress the possibility of a bidirectional relationship between adherence and HRQoL. On the one hand, adherence may enhance HRQoL by improving disease management and alleviating symptoms, such as pain. On the other hand, reduced adherence might result from symptom improvement (diminishing the perceived necessity for medication) or frustration from unrelieved symptoms despite adherence. Intriguingly, improved HRQoL itself might even contribute to non-adherence [36]. This dynamic may be better captured in longitudinal research that aims at modelling changes in adherence and health outcomes. Future longitudinal research and corresponding statistical analyses, designed to capture dynamic changes in adherence and health outcomes, may better illuminate this complex interplay, e.g. by applying cross-lagged models.

### Strengths and limitations

The study’s primary strengths lie in its longitudinal design, set in a real-world context, and the application of a comprehensive set of diverse and preselected factors with the potential to impact HRQoL in RA patients. This design enables a nuanced analysis of the specific influences of individual (sets of) predictors.

A limitation of the present study is that the sample is comprised exclusively of patients from Germany who were undergoing medical treatment at a single centre at the time of the study and who were willing to participate in a study on their adherence behaviour. It is therefore possible that the sample is not entirely representative of the general patient collective of individuals with RA, e.g. due to self-selection bias or regional peculiarities (e.g. the German health system). Looking at the population of RA patients in Germany, however, our sample does not deviate much. For instance, Albrecht *et al.* [37] summarize current data on rheumatological care in Germany, including ≈6000 RA patients. Patient characteristics are very similar to ours: 73% female (*vs* 69.5% in our sample), with a mean age of 63 years [s.d. 14; *vs* 60.2 (s.d. 13.4) in our sample] and an average duration of disease at assessment point of 14 months (s.d. 11; *vs*. 11 (s.d. 9) in our sample].

Second, results could be influenced by possible dropout bias [i.e. non-random dropout; dropout rates were 20% from baseline (*n* = 361) to T1 (*n* = 292) and 24% from T1 to T2 (*n* = 245)]. For instance, it might be that patients with higher pain, disease activity, depression or anxiety scores or lower adherence were more likely to drop out. However, dropout analysis (differences at baseline in scores between study sample and dropout sample at 12 months) revealed that only depression scores were slightly elevated in the dropout sample at baseline (*t*-test, HADS depression = 6.8 *vs* 5.7, *t* = 2.4, *P* = 0.019), whereas no group differences emerged for pain, disease activity, anxiety or adherence (*t*-tests and chi-squared tests, all *P*s > 0.17).

Furthermore, the SF-12 measure for HRQoL, while widely used, is not specifically tailored to RA and may lack sensitivity to certain critical aspects of RA, such as fatigue, which often constitutes a major stressor for RA patients ,with negative impacts on HRQoL [1, 38, 39]. In future studies, HRQoL measures tailored towards individuals with RA, such as the Rheumatoid Arthritis Impact of Disease (RAID) [40, 41] scale, might be a more suitable measure or used as a complementary measure. Finally, it is important to note that we only assessed obesity as a lifestyle factor, as other lifestyle factors, such as stress reduction or increased physical activity, have been identified as contributing factors to RA progression [42, 43]. These factors possess inherent value with regard to HRQoL, irrespective of their effect on RA.

A further limitation of our study is the reliance on patient self-reports in the absence of concurrent physician assessments, such as joint counts. Research shows that patient and physician assessments of disease activity often differ (i.e. discordance; e.g. Smolen *et al.* [44] and Bright *et al.* [45]). While the DAS28 is a validated measure of disease activity [46], discrepancies can arise. To provide a more comprehensive assessment, future research should adopt a hybrid follow-up model that combines remote patient-reported outcomes with periodic clinician assessments. This integrated approach would allow for a more thorough evaluation of patient health status, ensuring both patient perspectives and objective clinical findings are considered.

Another limitation is the broad categorization of medication use. While we assessed the effects of biologics (yes/no) on HRQoL, our analysis did not differentiate between classes of biologics, consider corticosteroid dosage or account for the duration of biologic therapy. The relatively small number of patients on biologics in our cohort limits the statistical power for such granular analyses. Future studies with larger patient populations should aim to conduct more detailed analyses of medication type, dosage and duration of use, which could provide a more nuanced understanding of their specific effects on HRQoL.

### Implications for research and practice

Given the chronic nature of RA, longitudinal studies with extended follow-up periods are essential. A 1-year follow-up, while informative, offers only a snapshot, particularly for patients already undergoing full treatment. Ideally, studies should include newly diagnosed RA patients at the onset of their condition, before initiating medical treatment, as exemplified by the Birmingham Early Arthritis Cohort (BEACON) [7] and follow long term. This way, changes in relevant variables can be investigated more thoroughly and, importantly, in long-term time periods. This will also allow accounting for bidirectional influences and long-term developments in the interplay between therapy, remission and adherence.

Second, even though we applied a comprehensive selection of predictor variables, further possible variables that might impact the HRQoL of individuals with RA are possible, especially behavioural factors such as (adherence to guidelines of) physical activity or sleep quality [47–49]. Researching these factors might be especially promising since these are at least partially modifiable behaviours.

Future research should also prioritize the development and evaluation of interventions aimed at improving HRQoL in RA patients. Such efforts should adopt a holistic perspective, assessing the full therapeutic regimen within real-world clinical settings. Beyond medication, critical factors such as patient–physician communication, health literacy and understanding of medication necessity, physical therapy, social support and mental health must be integrated into this framework. HRQoL should be evaluated as part of a multifaceted approach that includes multidisciplinary care programs and individual-specific factors [46, 50].

Our findings emphasize the need for clinicians to prioritize sustained pain management as well as psychological support, particularly addressing anxiety and depression, to improve both physical and mental HRQoL in RA patients.

## Conclusions

This study is the first comprehensive longitudinal analysis of a diverse array of HRQoL predictors in RA patients. Pain is identified as the most significant predictor of mental and physical HRQoL, even after a 12-month period, and should thus be accorded strong clinical attention. Age is strongly associated with (diminished) physical HRQoL, whereas anxiety and depression scores predict mental HRQoL. Other factors, such as adherence or lifestyle factors, were inconclusive. Future longitudinal studies investigating comprehensive sets of predictors of HRQoL and longer follow-ups in RA patients are needed to strengthen confidence in the analyses of predictors from cross-sectional (and short-term longitudinal) research.

## Supplementary Material

rkaf116_Supplementary_Data

## Data Availability

Data available on request. The data underlying this article will be shared on reasonable request to the corresponding author.
