# Supplementary material for: Predictors for health-related quality of life in patients with rheumatoid arthritis: a longitudinal study
Source: Rheumatol Adv Pract. 2025 Oct 9;9(4):rkaf116. doi: 10.1093/rap/rkaf116 (PMC12597878; doi:10.1093/rap/rkaf116)
Supplement: rkaf116_Supplementary_Data [file rkaf116_supplementary_data.zip › 25-075 Supplementary Figure S1.docx]

**Supplementary Figure S1:** Four histograms showing the distribution of physical and mental health-related quality of life scores


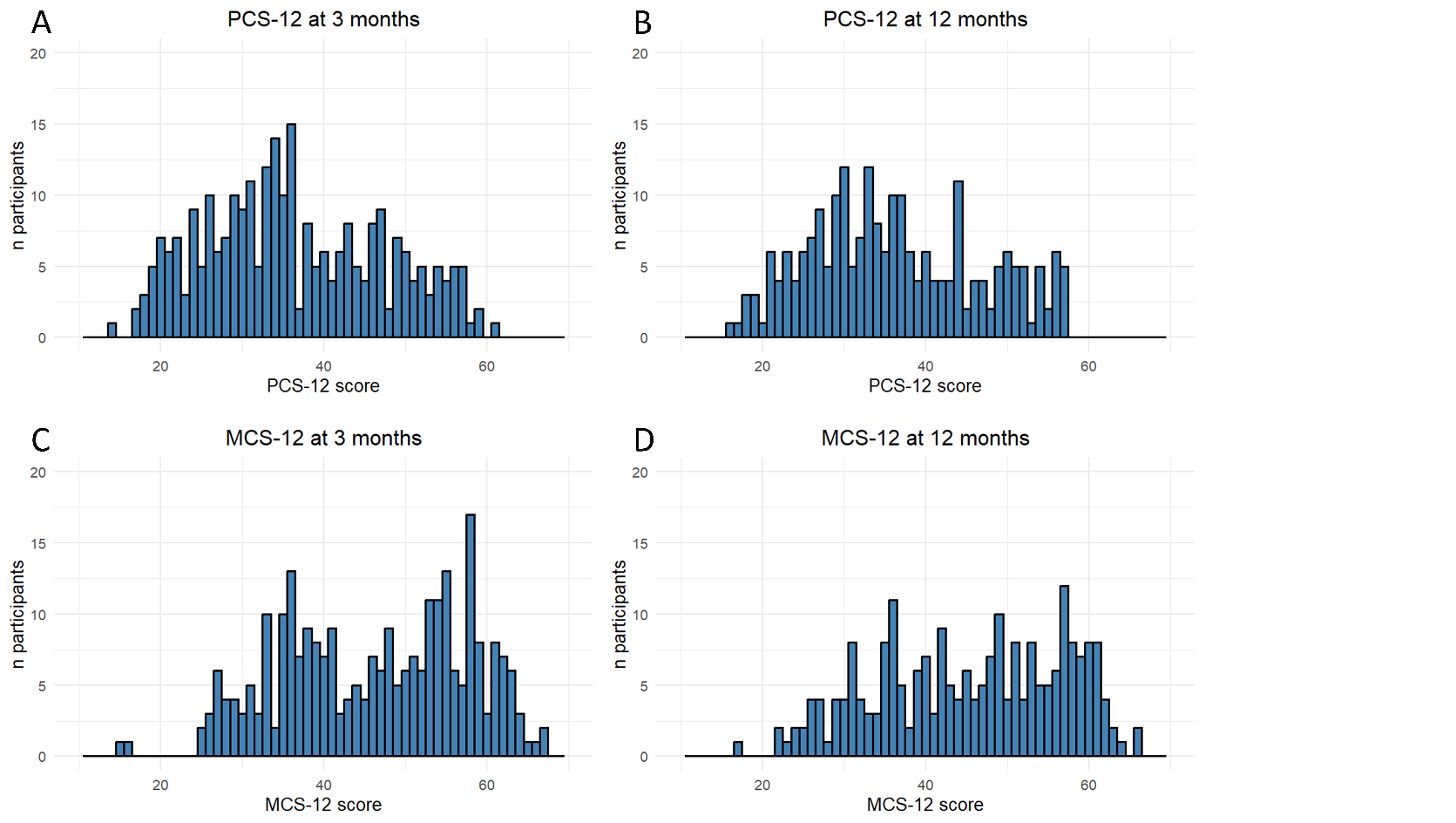


**Histogram for PCS12 and MCS-12 at 3 and 12 months follow-up;** *Note.* PCS -12 Physical Component Health-Related Quality of Life, MCS -12 Mental Component of Health-Related Quality of Life. **A:** PCS for 3 months follow-up, **B:** PCS for 12 months follow-up, **C:** MCS for 3 months follow-up, **D:** MCS for 12 months follow-up.

*ALT TEXT:*

**Fig 1**: Four histograms showing the distribution of physical and mental health-related quality of life scores for patients with rheumatoid arthritis at the three- and twelve-month follow-up periods. The mean scores for both physical and mental health were below the population mean and remained constant over time.
