# Supplementary material for: Predictors for health-related quality of life in patients with rheumatoid arthritis: a longitudinal study
Source: Rheumatol Adv Pract. 2025 Oct 9;9(4):rkaf116. doi: 10.1093/rap/rkaf116 (PMC12597878; doi:10.1093/rap/rkaf116)
Supplement: rkaf116_Supplementary_Data [file rkaf116_supplementary_data.zip › 25-075 Supplementary Data S1.docx]

**SUPPLEMENTARY DATA S1**

STROBE Statement—Checklist of items that should be included in reports of ***cohort studies***

|  | Item No | Recommendation |
| --- | --- | --- |
| **Title and abstract** | 1 | 1. Indicate the study’s design with a commonly used term in the title or the abstract   Title: “Predictors for health related quality of life in patients with rheumatoid arthritis (RA): A longitudinal study” |
|  |  | (*b*) Provide in the abstract an informative and balanced summary of what was done and what was found  Abstract provided, including methods (what was done) and results (what was found) |
| Introduction | | |
| Background/rationale | 2 | Explain the scientific background and rationale for the investigation being reported  Included in manuscript (“Background”) |
| Objectives | 3 | State specific objectives, including any prespecified hypotheses  Included in manuscript (“Background”) |
| Methods | | |
| Study design | 4 | Present key elements of study design early in the paper  Included in the “Methods” section (esp. sub-heading “study design and sample”) |
| Setting | 5 | Describe the setting, locations, and relevant dates, including periods of recruitment, exposure, follow-up, and data collection  Included in the “Methods” section (esp. sub-heading “study design and sample”) |
| Participants | 6 | (*a*) Give the eligibility criteria, and the sources and methods of selection of participants. Describe methods of follow-up  Included in the “Methods” section (esp. sub-heading “study design and sample”) |
|  |  | (*b*) For matched studies, give matching criteria and number of exposed and unexposed. *Not applicable.* |
| Variables | 7 | Clearly define all outcomes, exposures, predictors, potential confounders, and effect modifiers. Give diagnostic criteria, if applicable  Included in the “Methods” section, sub-headings “Measures” and “Statistics” |
| Data sources/ measurement | 8 | For each variable of interest, give sources of data and details of methods of assessment (measurement). Describe comparability of assessment methods if there is more than one group  Included in the “Methods” section, sub-headings “Measures” |
| Bias | 9 | Describe any efforts to address potential sources of bias  **Selection bias** –Selection bias is possible and discussed in the “strengths and limitations” section. A comparison is made between study sample and data on the population of RA patients in Germany to ascertain possible bias (cf. “Strengths and limiations”).  **Information bias** – The data of the present manuscript is based on self-report questionnaires. Thus, information bias might be an issue. It is difficult to check for this source of bias in a setting of self-report questionnaires sent by mail and filled in by participants at their home. To counteract possible information bias, we took care to use validated questionnaires (SF-12, BMQ, MARS, HADS, DAS 28; cf. section “Measures”). |
| Study size | 10 | Explain how the study size was arrived at  Included in the “Materials and Methods” section, sub-heading “study design and sample”: initial recruiting, inclusion criteria, and follow-ups are described. |
| Quantitative variables | 11 | Explain how quantitative variables were handled in the analyses. If applicable, describe which groupings were chosen and why  Included in the “Materials and Methods” section, sub-heading “Statistical analyses” |
| Statistical methods | 12 | (*a*) Describe all statistical methods, including those used to control for confounding  Included in the “Materials and Methods” section, sub-heading “Statistical analyses” |
|  |  | (*b*) Describe any methods used to examine subgroups and interactions  *Not applicable.* |
|  |  | 1. Explain how missing data were addressed   No explicit addressing of missing data (e.g. imputation). Only complete case analyses. |
|  |  | 1. If applicable, explain how loss to follow-up was addressed   No explicit addressing of loss to follow-up. |
|  |  | (*e*) Describe any sensitivity analyses  *Not applicable.* |
| Results | | |
| Participants | 13* | (a) Report numbers of individuals at each stage of study—eg numbers potentially eligible, examined for eligibility, confirmed eligible, included in the study, completing follow-up, and analysed  Included in the “Methods” section (esp. sub-heading “study design and sample”) |
|  |  | (b) Give reasons for non-participation at each stage  Reasons for non-participation, beyond not matching inclusion criteria, are unknown. |
|  |  | (c) Consider use of a flow diagram  Flowchart not included. Only three measurement points with comparatively small sample and no sub-group analyses. |
| Descriptive data | 14* | (a) Give characteristics of study participants (eg demographic, clinical, social) and information on exposures and potential confounders  Characteristics of study participants are included in the “Results” section, subheading “Sample characteristics and predictor variables”, including Table 2 “Sociodemographic and medical characteristics of study participants at Baseline (T0, N = 361) |
|  |  | (b) Indicate number of participants with missing data for each variable of interest  See “Results”, subheading “Descriptive results for main outcome”. Number of participants with missing data for each outcome variable is reported (“Rate of non-completed PCS / MCS measure for each measurement point[…]”) |
|  |  | (c) Summarise follow-up time (eg, average and total amount)  Follow-up time is Included in the “Materials and Methods” section (sub-heading “Study design and sample”) |
| Outcome data | 15* | Report numbers of outcome events or summary measures over time  Included in the “Results” section, esp. section “Descriptive results for main outcome” |
| Main results | 16 | (*a*) Give unadjusted estimates and, if applicable, confounder-adjusted estimates and their precision (eg, 95% confidence interval). Make clear which confounders were adjusted for and why they were included  *Unadjusted B coefficients are reported for individual variables in regression models (see Table 5 and Table 6).* |
|  |  | (*b*) Report category boundaries when continuous variables were categorized  *Not applicable.* |
|  |  | (*c*) If relevant, consider translating estimates of relative risk into absolute risk for a meaningful time period  *Not applicable.* |
| Other analyses | 17 | Report other analyses done—eg analyses of subgroups and interactions, and sensitivity analyses  *Not applicable.* |
| Discussion | | |
| Key results | 18 | Summarise key results with reference to study objectives  Included in “Discussion” section, subheading “Key Findings” |
| Limitations | 19 | Discuss limitations of the study, taking into account sources of potential bias or imprecision. Discuss both direction and magnitude of any potential bias  Included in “Discussion” section, subheading “Strengths and limitations” |
| Interpretation | 20 | Give a cautious overall interpretation of results considering objectives, limitations, multiplicity of analyses, results from similar studies, and other relevant evidence  Included in “Discussion” section, see e.g. subheadings “Interpretation, in relation to literature”, and “Implications for research and practice” |
| Generalisability | 21 | Discuss the generalisability (external validity) of the study results  Included in “Discussion” section, subheading “Strengths and limitations” |
| Other information | | |
| Funding | 22 | Give the source of funding and the role of the funders for the present study and, if applicable, for the original study on which the present article is based  Included at the end of manuscript (p. 19), along with ethical approval, disclosure, author contributions.  Ethical approval and consent to participate, Disclosure statement, Funding”, and author contributions are included at the end of the manuscript. |

**Note:** An Explanation and Elaboration article discusses each checklist item and gives methodological background and published examples of transparent reporting. The STROBE checklist is best used in conjunction with this article (freely available on the Web sites of PLoS Medicine at http://www.plosmedicine.org/, Annals of Internal Medicine at http://www.annals.org/, and Epidemiology at http://www.epidem.com/). Information on the STROBE Initiative is available at http://www.strobe-statement.org.
